# Judicial expertise in the post-pandemic era

**DOI:** 10.47626/1679-4435-2021-655

**Published:** 2021-12-30

**Authors:** Heinz Roland Jakobi, Alexandre Miguel

**Affiliations:** 1 Centro de Referência em Saúde do Trabalhador, Governo Estado de Rondônia, Porto Velho, RO, Brazil.; 2 2ª Câmara Cível, Tribunal de Justiça do Estado de Rondônia, Porto Velho, RO, Brazil.

**Keywords:** pandemic, COVID-19, social isolation, teleconsultation, pandemia, COVID-19, isolamento social, teleconsulta

## Abstract

**Introduction:**

In December 2019, the new coronavirus was recognized as the etiologic agent of a severe case of pneumonia in the city of Wuhan, China. The COVID-19 outbreak was initially defined by a series of epicenters and arrived in Brazil in February 2020, causing social isolation and suspension of on-site service provision, including in the Judiciary. There has been an exponential increase in lawsuits awaiting judicial expertise. The resumption of on-site judicial activities is a reality in the immediate post-pandemic, but it represents a major threat to Justice.

**Objectives:**

To propose a COVID-19 Contingency Plan specific for the environments of judicial expertise that indicates preventive measures complying with the provisions set forth by the health authorities to face the pandemic.

**Methods:**

We conducted a review of published Laws and Protocols of Standards and Conducts and developed a COVID-19 Contingency Plan for judicial investigations.

**Results:**

Opinions issued by the Brazilian Federal Council of Medicine contraindicate virtual medical expertise. The judicial expert assistance must return in person, but after ensuring the health protection of the participants of the Expert Act with the effective application of a COVID-19 Contingency Plan. Judicial experts have the duty of conforming to standards and ensuring the effective application of the Contingency Plan in the conduction of judicial investigations.

**Conclusions:**

The COVID-19 Contingency Plan has guidelines aimed at mitigating the risks of COVID-19 transmission in environments of judicial expertise. The Expert of the Court must be attentive to the dictates of the health authorities, adapting to the measures indicated promptly and immediately.

## Resumo

**Introdução:**

Em dezembro de 2019, o novo coronavírus foi reconhecido como agente etiológico de um grave quadro de pneumonia na cidade de Wuhan, na China. O surto da COVID-19 foi inicialmente definido por uma série de epicentros e chegou ao Brasil em fevereiro de 2020, provocando o isolamento social e a suspensão da prestação de serviços presenciais, inclusive no Poder Judiciário. Ocorreu um aumento exponencial de processos aguardando perícias judiciais. A retomada das atividades judiciais presenciais é uma realidade imediata do pós-pandemia, mas representa um grande e ameaçador desafio à Justiça.

**Objetivos:**

Propor um Plano de Contingenciamento da COVID-19 específico para os ambientes de perícias judiciais, no qual sejam indicadas as medidas de prevenção atendendo às determinações das autoridades sanitárias no enfrentamento da pandemia.

**Métodos:**

Realizamos uma revisão de Leis, Protocolos de Normas e Condutas publicadas, bem como procedemos à elaboração de um Plano de Contingenciamento da COVID-19 para perícias judiciais.

**Resultados:**

Pareceres do Conselho Federal de Medicina contraindicam a realização de perícia médica virtual. O atendimento pericial judicial deverá retornar de forma presencial, mas depois de assegurada a proteção à saúde dos participantes na perícia com a efetiva aplicação de um Plano de Contingenciamento da COVID-19. Os peritos judiciais têm por dever de ofício observar as normas e a efetiva aplicação do Plano de Contingenciamento na realização das perícias judiciais.

**Conclusões:**

O Plano de Contingenciamento da COVID-19 possui as orientações que visam à mitigação dos riscos de transmissão da COVID-19 em ambientes de perícias judiciais. O Perito do Juízo deve estar atento aos ditames das autoridades sanitárias, adequando-se às medidas indicadas de forma pronta e imediata.

## INTRODUCTION

In December 2019, the new coronavirus (severe acute respiratory syndrome coronavirus 2 [SARS-CoV-2]) was recognized as the etiological agent of a severe case of pneumonia in the city of Wuhan, China. The SARS-CoV-2 virus, which causes the new coronavirus disease (COVID-19), was identified in 2020 as being a “relative” of the SARS-CoV virus. The SARS-CoV-2 is highly infectious and, in severe cases, generates a cytokine storm, due to an excessive reaction of the immune system to the virus.^1,2^

SARS CoV-2 is transmitted from human to human through contact with respiratory droplets (cough, sneeze, catarrh) of saliva, coming from people infected with the virus or through contact with contaminated surfaces after contact with mouth, nose, and eyes.

According to the World Health Organization (WHO),^1-3^ people at high-risk, i.e., who are considered vulnerable to the SARS-CoV-2, are those who meet some of the following conditions:

I - age 60 years or older;

II - insulin-dependent diabetes;

III - chronic renal failure;

IV - chronic obstructive pulmonary disease (COPD), pulmonary emphysema, moderate or severe asthma, active tuberculosis, or pulmonary sequela of tuberculosis;

V - severe heart diseases, heart failure, and severe systemic arterial hypertension;

VI - immunosuppressed patients, except those with autoimmune diseases not using immunosuppressive agents;

VII - morbid obesity, body mass index (BMI) greater than or equal to 40;

VIII - cirrhosis or liver failure;

IX - pregnant women or breastfeeding mothers of children younger than 1 year;

X - caregivers of or those who live with one or more individuals with confirmed diagnosis of COVID-19 infection.

The signs and symptoms of COVID-19^1-3^ are fever (> 37.8ºC); cough; dyspneia; myalgia and fatigue; upper respiratory tract symptoms; gastrointestinal symptoms such as diarrhea (rarer) and anosmia, which have been frequent complaints. The typical clinical presentation of a flu syndrome may range from asymptomatic or mild symptoms to worsened clinical presentations.

## INSTITUTIONAL ACTS OF DISEASE MITIGATION

The COVID-19 outbreak was initially defined by several changing epicenters, including the city of Wuhan, China, Iran, northern Italy, Spain, and the city of New York. After SARS-CoV-2 arrived in Brazil, on February 6, 2020, Law no. 13,979 was promulgated,^4^ which regulates the “measures to face the public health emergency of international concern resulting from the coronavirus responsible for the COVID-19 outbreak.” In its article 3º, § 8º, this Law explains that “the measures set forth in this article, when adopted, shall safeguard the execution and functioning of public services and essential activities.”^1,2,5^

Belatedly, only on March 11, 2020, the WHO defined the outbreak caused by COVID-19 as a pandemic - infectious disease that spreads throughout the population from a wide geographical region, such as a continent or even the entire planet.^1,6^

The Federal Decree no. 10,282, of March 20, 2020,^7^ considers the following activities as public services and essential activities (3^rd^ article, 1^st^ §): “medical expertise activities related to social security, covered in article 194 of the Constitution” (item XXXIII) and “medical expertise activities related to characterization of physical, mental, intellectual, or sensory impediment of people with a disability, by integrating multiprofessional and interdisciplinary teams, in order to recognize rights provided by law” (item XXXIV).

On March 17, 2020, the Higher Council of Labor Justice issued the Act CSJT.GP.SG no. 047/2020,^8^ which, in its article 10, “temporarily suspends public visitation and on-site services to the external public that may be provided online or by telephone.” On the same day, the Higher Labor Court (Tribunal Superior do Trabalho, TST) suspended the on-site provision of services in the TST with regard to non-essential activities through Act no. 126/GDGSET.GP;^9^ furthermore, the Regional Labor Court of the 14^th^ Region edited Act no. 002/2020/TRT14/GP^10^ on emergency and temporary measures to prevent contagion with SARS-CoV-2. Thus, it regulates permission for temporary, extraordinary remote work to judges, civil servants, and employees in general.

In turn, Resolution no. 313,^11^ issued by the National Justice Council (Conselho Nacional de Justiça, CNJ) on March 19, 2020, establishes, in the Judiciary, the “regime of Extraordinary On-Duty Services, in order to standardize the operation of judicial services, with the purpose of preventing contamination with the new coronavirus - (COVID-19) and ensuring access to Justice in this emergency period,” stating, in its 2^nd^ article, § 1^st^, item II, that “among the minimum essential services, there the maintenance of services related to the issuance and publication of judicial and administrative acts.” Moreover, the 2^nd^ article, § 2^nd^, provides that “the directors of essential services and activities shall organize a method of service provision that is primarily in a remote manner,” and the 6^th^ article states that “courts may regulate the holding of virtual meetings.”

On March 18, 2020, Act no. 003/2020/TRT14/GP,^12^ issued by the Regional Labor Court of the 14^th^ Region, revoked Act no. 002/2020/TRT14/GP,^10^ of March 17, 2020, disposing, in context of the Regional Labor Court of the 14^th^ Region, on “emergency and temporary measures to prevent contagion with the new coronavirus that causes COVID-19”, and its 2^nd^ article, item V, provides that “judicial expertise activities are exceptionally and preventively suspended.”

On March 19, 2020, Joint Act CSJT.GTP.VP and CGJT no. 001^13^ was edited by the Higher Council of Labor Justice, which suspends “the on-site provision of services in the 1^st^ and 2^nd^ degree Labor Justice and establishes a protocol for the minimum and restricted provision of essential services to comply with the attributions of the 1^st^ and 2^nd^ degree Labor Justice as an emergency measure to prevent the spread of the new coronavirus (COVID-19)”.

Federal Decree no. 10,282,^14^ of March 20, 2020, provides the following activities as public services and essential activities (3^rd^ article, 1^st^): “medical expertise activities related to social security, covered in article 194 of the Constitution” (item XXXIII) and “medical expertise activities related to characterization of physical, mental, intellectual, or sensory impediment of people with a disability, by integrating multiprofessional and interdisciplinary teams, in order to recognize rights provided by law” (item XXXIV).

Provisional Executive Order no. 927,^14^ enacted on March 22, 2020, “disposes on labor measures to face the state of public disaster recognized by Legislative Decree no. 6, of March 20, 2020, and the public health emergency of international concern resulting from coronavirus (COVID-19), and sets forth other provisions.”

Act no. 004/2020/TRT14/GP,^15^ enacted on March 23, 2020, “provides new measures, in the setting of the Regional Labor Court of the 14^th^ Region, to prevent contamination by the new coronavirus that causes COVID-19, and conforms to the guidelines outlined by the CNJ in Resolution no. 313,^11^ of March 29, 2020 and by the CNJ in the Joint Act CSJT.GTP.VP and CGJT no. 001,^13^ of March 19, 2020.”

In the context of the Federal Justice of the 1^st^ Region of the Court, Judicial Districts and Sub-districts, Resolution PRESI no. 9953729,^16^ issued on March 27, 2020, defined the temporary measures to face the pandemic, “instituting Virtual Trials for electronic lawsuits in the electronic lawsuits system and On-site Meetings with Video Support, contemplating fractional courts, special court, districts, and chambers.”

In Rondônia, Decree no. 24,979,^17^ of April 26, 2020, declared “State of Public Disaster in the entire state.” Furthermore, State Decree no. 25,049,^18^ of May 14, 2020, instituted the Controlled Social Distancing System in order to prevent and face the epidemic caused by the new coronavirus - COVID 19”. In the state of Rondônia, the declaration of state of public disaster was reaffirmed in the entire state and Decree no. 24,979,^17^ of April 26, 2020, was revoked. However, activities of “hospitals, health clinics, dental clinics, clinical analysis laboratories, and drugstores” were not suspended, as provided in subparagraph d of the Appendix.

CNJ Resolution no. 317,^19,20^ of April 30, 2020, disposes on the “conduction of expertise activities by electronic or virtual means in actions that discuss pension benefits, either disability or welfare ones, for the duration of the effects of the crisis resulting from new coronavirus pandemic, and sets forth other provisions.”

On May 19, 2020, the CNJ Crisis Committee issued an opinion in the context of the management control procedure no. 0003451-62.2020.2.00.0000,^21^ which concluded in favor of feasibility of “practicing on-site expertise at doctor’s offices, provided that severe conditions are met, including the following: a) indispensability of expertise to be performed on-site; b) thorough assessment of local situation with regard to pandemic evolution and social distancing regulations; c) strict compliance with provisions related to sanitary and hygiene measures and alike.”

With the suspension of judicial expertise activities, even in an extraordinary and preventive manner, there was an exponential growth in the number of lawsuits waiting for scheduling of examinations in all courts throughout the country.

The 5^th^ article of the 1988 Federal Constitution, in its LXXVIII item,^22^ ensures “the reasonable duration of legal lawsuits and the means that ensure the agility of their processing.” Therefore, in view of the essential nature of the jurisdictional activity and the need to ensure minimum conditions for its continuity, aligning this activity with the preservation of the health of judges, public agents, lawyers, and users in general, there is an urge to tackle the great restrained demand of proceedings awaiting judicial expertise.

Several alternatives have been proposed, in which actors’ assistance could be provided by “remote or virtual” judicial expertise, using the new communication technologies, in accordance with telemedicine,^23^ a service that was already laid down in the 3^rd^ Article of Resolution CFM 1643/2002,^24^ which disposed “on urgency and emergency care through Telemedicine,” and in Ordinance MS 467/2020^25^ during the pandemic. This type of assistance is also known as “tele-expertise, teleconsultation, or only technical expertise” and was then discussed in several judicial and legal institutions and by Brazilian physicians.

However, Opinion no. 03/2020, issued by the Federal Council of Medicine (Conselho Federal de Medicina, CFM),^26^ and Resolution 10/2020,^27^ of May 18, 2020, suggest that the practice of virtual medical expertise and simplified technical expertise may break the Medical Ethics Code. Medical experts expressed the unfeasibility of practicing these activities in a telematic manner, since “it is not permitted to perform occupational medical examinations with telemedicine resources, without conducting a direct clinical examination of the worker,” as stated in CFM Opinion 8/2020.^28^

On July 24, 2020, the Federal Special Court, by means of the Judiciary District of Rondônia, enacted Ordinance no. 10587376,^29^ which “regulates the practice of medical expertise in experts’ offices during the COVID-19 pandemic.”

Finally, courts must proceed with on-site judicial expertise, in order to tackle the huge and growing accumulation of lawsuits, ensuring the reasonable duration of lawsuits and the means to ensure the agility of their processing.

The resumption of on-site judicial expertise is an immediate reality of the post-pandemic era. However, in order to preserve the health of the actors involved in the production of judicial expert evidence, it is important to have a well-founded COVID-19 Contingency Plan (CCP),^3^ designed by Courts and effectively applied by experts, lawyers, lawsuit parties, and their companions.

The CCP^3^ specific for environments of judicial expertise, should recommend the adoption of measures to prevent the spread of SARS-CoV-2, complying with provisions, procedures, and practices indicated by health authorities to face the COVID-19 pandemic.

Below is a list of the basic prevention and control measures that should be included in a CCP to face COVID-19 in the context of judicial expertise.

### PROTOCOL OF HYGIENE-SANITARY STANDARDS AND PROCEDURES:

- A punctual time schedule should be established in order to avoid crowding.
- In the Judicial Act, only the Claimant/Plaintiff of the judicial investigation is allowed to be present, with no companions, except for minors, those incapacitated due to mental disorders, or people with locomotion difficulties, and for medical experts providing technical assistance for the parties.
- Experts, expert assistants, claimants, and possible companions involved in the expertise investigation should strictly comply with the sanitary and health guidelines issued by public authorities.
- Participants should not attend medical expert investigation if they have signs or symptoms of flue syndrome.
- No participant of the Expert Act may present with “flu symptoms or fever.” If any participant is symptomatic, the expertise is rescheduled to a day no sooner than 15 days.
- Visual warnings with guidance on flows, mandatory mask wearing, hand sanitation sites, and respiratory etiquette should be affixed.
- It is indicated to affix posters with instructions of hygiene, hand washing technique, etiquette of cough, sneezing, and nasal secretion, and the remaining strategies to prevent contagion, reinforcing visual communication in all environments.
- Social etiquette for cough and sneezing should be maintained.
- Experts should administer a previous clinical questionnaire, either written or verbal.
- Experts should identify and measure the temperature of all participants and, if fever or any other suspected symptom of COVID-19, the individual presenting with these symptoms shall not participate in the expertise act.
- If the Claimant have signs and symptoms of the disease, the Expertise Act should be postponed for 14 days and the Claimant, now a patient, should undergo examinations in the Public Health Network and remain in household isolation under medical treatment.
- All those present should mandatorily respiratory masks during the entire medical judicial act, and refusal to wear personal protection equipment constitute reason for not conducting expertise work.
- Experts should ensure a proper environment for conducting expertise work, with sanitized restrooms, and ensuring safe distancing between people, in order to avoid crowding, especially in spaces without proper ventilation.
- The ventilation rate should be increased in work environments, either by natural or artificial means, in order to improve air exchange in the place.
- A minimum distancing of 2 meters is ensured entre the participants of expertise.
- Seats/chairs/tables and the floor should be marked with strips/tapes in order to maintain the minimum distancing of 2 meters.
- A dispenser with paper towel and liquid soap should be made available for hand sanitation, or a foot-operated hand sanitizer station 70% alcohol gel.
- Guidance should be provided on frequent hand hygiene with water and soap, as well as on situations when the use of alcohol gel is indicated and on necessary precautions to prevent accidents with the product.
- Physical barriers, such as polycarbonate or transparent glass protectors, should be installed on the consultation desk.
- Doctors’ offices should have washbasins and be well ventilated, preferably by natural means.
- Negative pressure ventilation systems should be installed in some situations, such as in procedures that generate aerosols (e.g., isolation beds in health institutions and autopsy rooms in mortuary environments).
- It is important to reinforce cleaning and disinfection procedures with disinfectants in all environments, surfaces, and devices, at least in the beginning and in the end of activities.

### ON VESTMENTS AND PROCEDURES

#### Of experts and their assistants

- Ensuring respiratory containment - surgical mask or PFF2/N95 respiratory protection mask;
- Wearing gloves, googles, or face shield and disposable long sleeve woven or non- woven gowns.
- Ensuring to install a panel made of glass or other material between the attendant and the worker/patient, whenever possible, or wearing a face shield;
- Measuring the temperature of all individuals participating in the expert investigation;
- Washing hands with soap frequently;
- Wearing disposable procedure gloves;
- Cleaning and disinfecting highly touched objects and surfaces using sodium hypochlorite or 2% biguanide.

#### Of claimants and their companions

- Maintaining a minimum distance of 2 m between people;
- Wearing a mask for personal use - cloth or surgical.

## Conclusion

This article provides basic guidance on prevention, control, and mitigation of risks of contamination with COVID-19 in environments of judicial expertise in Courts and public and private doctor’s office. Very specific situations may occur, which judicial experts should contemplate in a personal and individualized CCP.

We also warn that judicial experts have the duty of conforming to sanitary standards when developing the CCP and ensuring its effective application in conducting judicial investigations.

Since COVID-19 is a little understood disease that has not been effectively controlled yet, the clinical and sanitary process becomes very dynamic, always presenting new paradigms. The Expert of the Court must be attentive to the dictates of the Brazilian health authorities and adapt the “applicable measures indicated promptly and immediately.”
